# Social relationship correlates of major depressive disorder and depressive symptoms in Switzerland: nationally representative cross sectional study

**DOI:** 10.1186/1471-2458-14-273

**Published:** 2014-03-24

**Authors:** Steven D Barger, Nadine Messerli-Bürgy, Jürgen Barth

**Affiliations:** 1 Department of Psychology, Northern Arizona University, PO Box 15106, Flagstaff, AZ 86011, USA; 2 Department of Psychology, Clinical Psychology and Psychotherapy, University of Bern, Fabrikstrasse 8, Bern 3012, Switzerland; 3 Institute of Social and Preventive Medicine, University of Bern, Finkenhubelweg 11, Bern 3012, Switzerland; 4 Institute for Complementary and Integrative Medicine, University of Zurich, Switzerland, Raemistrasse 100, 8006 Zurich, Switzerland

**Keywords:** Depression, Social networks, Support, Social, Social isolation, Swiss Health Survey

## Abstract

**Background:**

The quality and quantity of social relationships are associated with depression but there is less evidence regarding which aspects of social relationships are most predictive. We evaluated the relative magnitude and independence of the association of four social relationship domains with major depressive disorder and depressive symptoms.

**Methods:**

We analyzed a cross-sectional telephone interview and postal survey of a probability sample of adults living in Switzerland (*N* = 12,286). Twelve-month major depressive disorder was assessed via structured interview over the telephone using the Composite International Diagnostic Interview (CIDI). The postal survey assessed depressive symptoms as well as variables representing emotional support, tangible support, social integration, and loneliness.

**Results:**

Each individual social relationship domain was associated with both outcome measures, but in multivariate models being lonely and perceiving unmet emotional support had the largest and most consistent associations across depression outcomes (incidence rate ratios ranging from 1.55-9.97 for loneliness and from 1.23-1.40 for unmet support, *p*’s < 0.05). All social relationship domains except marital status were independently associated with depressive symptoms whereas only loneliness and unmet support were associated with depressive disorder.

**Conclusions:**

Perceived quality and frequency of social relationships are associated with clinical depression and depressive symptoms across a wide adult age spectrum. This study extends prior work linking loneliness to depression by showing that a broad range of social relationship domains are associated with psychological well-being.

## Background

Depressive disorders affect about one fifth of the population over the life course [1,2] and are associated with impairments in daily functioning and work [3]. Major depression ranks fourth among disorders with the highest burden of disease worldwide, and it is expected to be ranked first in high-income countries by 2030 [4]. Subclinical depressive symptomatology is also important because it is more prevalent than major depression [5] and is associated with increased health care utilization [6] as well as social and work-related impairments [7,8]. There are a number of established predictors of clinical depression and depressive symptomatology including critical life events [9,10], socioeconomic status [11], and inadequate social relationships [12]. The present study explored the association of social relationships with major depressive disorder and subclinical depressive symptoms in a representative sample of Swiss adults.

Because social connectedness is central to human existence [13,14], social relationship deficits are expected to elicit distress and depression [14-17]. Consistent with this perspective, numerous social relationship domains show an inverse association with depression and depressive symptoms [18-26] but few of these studies have used a broad, theoretically representative set of social relationship domains. Identifying the specific social relationship domains that are most strongly associated with depression and depressive symptoms will advance theory as well as provide a foundation for intervention.

*Social support* and *social engagement* represent two broad social relationship domains which are each comprised of a number of dimensions. Social support, representing material and psychological resources that facilitate one’s ability to cope with stress [27], encompasses opportunities for venting feelings (*emotional support*) and assistance with daily tasks (*instrumental support*) [27]. Social engagement [15], also called social integration [27], reflects participation in a wide range of social relationships such as getting together with friends or family, being married, attending social functions, etc. Overlapping these domains is *loneliness*, which represents the perception that the number of one’s social relationships is smaller than desirable or that desired intimacy with others has not been achieved [28].

Measures of support and social integration are each associated with depressive symptoms [18,24,25,29,30] and loneliness appears to be a particularly potent predictor of depressive symptoms both cross-sectionally and prospectively [19,31-33]. Given this growing evidence, our study addresses the need to simultaneously evaluate different social relationship resources in one analysis (e.g., [24,26]). This approach is important to establish, for example, whether the subjective components of isolation (e.g., loneliness, perceived support) are distinct from more objective features such as the frequency of participation in social activities [26]. Utilizing this approach with assessments of both depressive disorder and subclinical depressive symptoms will illuminate which social relationship resources are most important and whether social relationship determinants are consistent across clinical and non-clinical mental health contexts.

Another gap in the social relationships and depression literature is that it is based upon fairly narrow demographic groups such as college students [18], small urban samples of older adults [19,22], inpatients [20] or larger samples of predominantly elderly participants [21,24,26] (see [23,25,34,35] for exceptions). Population-based data are necessary to 1) accurately characterize the form and extent of the social relationships/depression association; and 2) to ensure inclusion of the age group with the largest depression burden (persons aged 18–49) [36,37].

The present study examined the association of four social relationship domains with both major depression and depressive symptoms in a large representative sample of Swiss adults 18–99 years of age. We included measures of emotional support (availability of a confidant; unmet social support needs), tangible support, social integration (social contact frequency and marital status) and loneliness. Twelve-month major depressive disorder was assessed using the Composite International Diagnostic Interview short form (CIDI-SF; [38]), a fully structured assessment conducted by trained lay interviewers via telephone. Depressive symptoms were assessed with the Depression Screening Questionnaire, a 10-item written measure [39] obtained in a follow-up postal survey.

Given prior research we expected that all social relationship domains would be associated with both depression outcomes in bivariate analyses. Extant literature also suggests that loneliness should have a large association with both depression measures, but given the rarity of similar studies jointly comparing the remaining social relationship domains we did not make specific predictions regarding which social relationship variables would have the strongest association with the depression outcomes.

## Methods

### Participants

The Swiss Health Survey is a periodic survey conducted by the Swiss Federal Office of Statistics. We utilized data from the 2007 Swiss Health Survey which included a representative sample of persons living in Switzerland. A random sample from an address pool of phone registered individuals was used for data collection. Four criteria had to be fulfilled to be included in the survey: a) living in Switzerland for more than three months, b) living in a private household, c) being older than 15 years, and d) speaking at least one of the three interview languages (German, French or Italian). Data collection was divided into four assessment periods to control seasonal influence.

Individuals were contacted by telephone and asked to participate in a telephone interview and secondly to complete a written questionnaire which was mailed to them after the initial interview. Most interviews were conducted by telephone although a face-to-face interview was offered in some cases (i.e., age > 74 years, severe somatic disease or accident or language difficulties). The data collection and data storage for the Swiss Health Survey (Schweizerische Gesundheitsbefragung) does not require formal approval by an ethical committee. This data collection is specifically permitted under Swiss law (Verordnung über die Durchführung von statistischen Erhebungen des Bundes vom 30. Juni 1993 (SR 431.012.1) and Verordnung über die eidgenössische Volkszählung vom 19. Dezember 2008 (SR 431.112.1)). Individuals invited to participate received a brief description of the study and could decline to participate or withdraw at any time. Participants’ responses were treated confidentially and aggregated anonymous responses were utilized for analyses presented herein. This secondary analysis of anonymous archival data was approved by the Institutional Review Board of Northern Arizona University.

A total of 18,760 individuals (66% of those contacted) took part in the interview and 77% (14,393) of those interviewed returned the written questionnaire. Census-based weights for the postal survey participants were used to represent the Swiss population. We restricted our analyses to postal survey participants 18 years or older (*N* = 14,016) who had no missing values for the depressive symptoms questionnaire (*N* = 12,286).

### Measures

*Major depressive disorder* was assessed by using a short version of the Composite International Diagnostic Interview (CIDI-SF). The CIDI-SF is a fully structured diagnostic interview administered over the phone by trained lay interviewers. The CIDI-SF uses DSM-III-R [40] criteria to classify a major depressive episode in the last twelve months. Validation studies report good sensitivity and specificity with the full CIDI (89,6% and 93,9%, respectively) [38].

*Depressive symptoms* were assessed with the 10-item self-administered Depression Screening Questionnaire (DSQ) [39]. Items assess depressed mood, loss of interest, fatigue, weight change, sleep difficulties, slower movement or speech, decreased sexual desire, feeling worthless or guilty, trouble concentrating and thoughts of death or suicide over a period of the last two weeks using response options that range from *no, never* (0), to *sometimes* (1) or *on most days* (2). Scores can range from 0–20. Cronbach’s alpha for the DSQ in this sample was 0.82. Unlike some depressive symptom measures [41] the DSQ contains no loneliness items and thus is free of potential predictor-criterion overlap.

*Social relationships* were assessed with measures of social support and social integration. These items were developed specifically for this survey and capture a broad range of social relationship resources. Social support was represented by having a *confidant* (one item; “Among the people you are close to is there someone with whom you can always talk about really personal problems? No one, one person, many people”), having *unmet support needs* (one item; “Do you ever miss having someone to talk to about really personal problems?” Yes, no) and having *tangible support* (one item; “Do you have a family member or a neighbor you can always ask to help or support you with your daily activities?” *No one*, *one person*, *many people*). Social integration was assessed with two variables, *marital status* (one item; married/cohabiting vs. other) and the frequency of *social contacts* (five items). Social contact items included the frequency of meeting or phoning friends or relatives and participating in group activities. Respondents were asked: How often do you meet with friends or acquaintances at your home or theirs? How often do you meet with family members at your home or theirs? How often do you call or receive calls from your family members? How often do you call or receive calls from your friends or acquaintances? Response options for these items included *Almost never or never* (*less than once per year*); *at least once a year*; *at least once a month*; *at least once a week*; *daily or almost daily*. Frequency of participation in group activities or clubs (*never*, *rarely*, *a few times a year*, *about once a month*, *about once a week* and *almost daily*) was assessed with the question “How often do you participate in the activities of an association, club, political party or any other type of group?” These ordinal responses were coded 0–4 (0–5 for the group activities item) and summed and recoded into five categories for analysis (summary scores of 0–9 [referent], 10–12, 13–14, 15–16, and 17–21). This ordinal coding is comparable with other studies [19,26]. The small cell sizes (range 6–50; *M* = 23) for the lowest contact frequency categories (0–4) precluded reliable comparisons across these groups.

*Loneliness* was assessed with one item asking how often the respondent felt lonely (never, sometimes, quite often, very often). Because only 1% of the sample endorsed the “very often” category we merged this category with “quite often” to obtain more stable estimates.

To facilitate comparisons among social relationship regression coefficients we scaled confidant and tangible support to have a 0–1 range, i.e., instead of values of 0, 1 or 2 they were coded 0, 0.5, and 1.0 [42]. Ratio measures of association tend to be larger for binary versus polytomous variables and this scaling provides a more equal context for comparison of social relationship regression coefficients [42].

*Sociodemographic variables* included age, gender, education level (three indicator variables representing four education categories; mandatory, secondary, tertiary/gymnasium, university), nationality (Swiss, Italian, other) and the predominant language spoken in the respondent’s region (German, French, or Italian).

### Data screening and analysis

Among the three social relationship variables with more than two categories (loneliness, confidant, tangible support) loneliness did not meet an interval assumption in bivariate analyses [43]. Therefore we utilized two indicator variables to represent three levels of loneliness.

The distribution of depressive symptoms violated ordinary least squares (OLS) regression assumptions, e.g., symptoms were positively skewed and the variance was greater than the mean. We therefore analyzed the DSQ using negative binomial regression, exponentiating the coefficients to obtain incidence rate ratios (IRR). This approach preserves the interval properties of the scale and accommodates skewness and overdispersion [42]. Untransformed regression coefficients and statistical conclusions from negative binomial regressions were very similar to OLS regressions with square-root transformed DSQ values.

We estimated IRRs for binary major depressive disorder directly using a general linear model with a Poisson distribution and a log link function between covariates and the outcome. Robust standard errors were estimated to provide appropriate coverage for the confidence intervals [44]. The IRR, which reflects the ratio of incidence for the exposed group divided by the unexposed group, is the most general measure of association [45], provides accurate point and interval estimates [46] and is preferred to the odds ratio obtained from logistic regression [47,48]. The magnitude of the IRR point estimate was generally smaller than the corresponding odds ratio but the direction and significance was almost identical to coefficients obtained from logistic regression (data not shown).

In two parallel sets of analyses we regressed major depressive disorder or depressive symptoms on social relationships. First, we entered every social relationship variable separately. This provided an estimate of the strength of association for each individual social relationship indicator. Second, we simultaneously entered all social relationship variables to determine their independence and to estimate which had the strongest association when adjusting for the others. In the third step we entered indicator variables representing age, gender, education, regional language, and nationality to determine whether these sociodemographic factors altered the association of social relationship variables with the depression outcomes. Analyses were considered statistically significant if the two-tailed *p*-value was less than or equal to 0.05. All analyses were conducted with Stata 11.2 (Stata Corp., College Station, Texas USA) and variance estimates incorporated the survey design, i.e., sampling strata and weights. Survey-based IRR’s were slightly different from those calculated directly from the unweighted frequencies shown in Table 1.

**Table 1:** Demographic, social and affective characteristics of Swiss health survey participants, 2007

		**Major depressive disorder**	
	**Total *N*** **= 12,286)**	**Absent (*n*** **= 11,760)**	**Present (*n*** **= 526)**
Age, *M* (SD)	49.1 (16.4)	49.3 (16.5)	45.2 (14.2)
Percent female (*N*)	54.1 (6643)	53.6 (6300)	65.2 (343)
Nationality, percent (*N*)			
Swiss	89.5 (10,099)	89.7 (10,553)	84.8 (446)
Italian	2.2 (267)	2.1 (246)	4.0 (21)
Other	8.3 (1018)	8.2 (959)	11.2 (59)
Missing	.02 (2)	.02 (2)	
Regional language, (*N*)			
German	63.0 (7739)	63.2 (7430)	58.8 (309)
French	29.7 (3645)	29.5 (3472)	32.9 (173)
Italian	7.3 (902)	7.3 (858)	8.4 (44)
Education, percent *(N)*			
Mandatory	9.5 (1168)	9.3 (1098)	13.3 (70)
Secondary	60.5 (7427)	60.5 (7110)	60.3 (317)
High school	9.2 (1129)	9.2 (1087)	8.0 (42)
University	20.9 (2562)	21.0 (2465)	18.4 (97)
Marital status, percent (*N*)			
Single/divorced/widowed/separated	44.8 (5500)	44.1 (5186)	59.7 (314)
Married/cohabiting	55.2 (6777)	55.8 (6565)	40.3 (212)
Missing	.07 (9)	.07 (9)	
Lonely (how often)			
Never	69.3 (8511)	70.9 (8333)	33.8 (178)
Sometimes	27.1 (3333)	26.5 (3118)	40.9 (215)
Quite often/very often	3.5 (430)	2.5 (297)	25.3 (133)
Missing	0.1 (12)	0.1 (12)	
Confidant (someone to talk to about problems?)			
No one	4.5 (551)	4.2 (497)	10.3 (54)
One person	24.3 (2981)	24.2 (2846)	25.7 (135)
Many people	71.0 (8728)	71.4 (8392)	63.9 (336)
Missing	0.2 (26)	0.2 (25)	0.2 (1)
Unmet support (do you ever miss having someone to talk to about your problems?)			
No	80.3 (9861)	81.2 (9547)	59.7 (314)
Yes	19.5 (2398)	18.6 (2189)	39.7 (209)
Missing	0.2 (27)	0.2 (24)	0.6 (3)
Tangible support (do you have family or a neighbor to help and support you with daily activities?)			
No one	6.3 (778)	6.0 (704)	14.1 (74)
One person	9.9 (1212)	9.8 (1151)	11.6 (61)
Many people	83.1 (10209)	83.5 (9821)	73.8 (388)
Missing	0.7 (87)	0.7 (84)	0.6 (3)
Social contacts quintile			
0-9	12.3 (1514)	12.1 (1424)	17.1 (90)
10-12	28.6 (3511)	28.5 (3347)	31.2 (164)
13-14	25.2 (3091)	25.1 (2949)	27.0 (142)
15-16	21.2 (2610)	21.6 (2534)	14.5 (76)
17-21	12.7 (1560)	12.8 (1506)	10.3 (54)
Depressive symptoms, *M* (SD)	3.4 (3.2)	3.2 (2.9)	7.9 (4.7)

Participants included those who responded to both the telephone survey and to the written questionnaire and who had no missing data for the depression scales. Twelve-month major depressive disorder was assessed with the Composite International Diagnostic Interview short form [38]. Depressive symptoms experienced over the last two weeks were assessed with the Depression Screening Questionnaire [39]. Social contacts represent the sum of 5 questions. Four contact items encompassed a) meeting or phoning b) friends or relatives. The fifth question assessed participation in group activities such as clubs or organizations. Contact items were anchored with *never* (0), *rarely* (1), *twice a year* (2), *once a month* (3), *once a week* (4) and *almost daily* (5) response options. Response options for group activities included *never (0)*, *rarely (1)*, *a few times a year (2)*, *about once a month (3)*, *about once a week (4)* and *almost daily (5). M* = mean; SD = standard deviation.

## Results

Postal survey respondents who were not included in the analyses were more likely to be female, older, less educated, unmarried, and to report “other” nationality compared to those in the analytic sample (data not shown). Descriptive statistics for the analytic sample are provided in Table 1. The estimated 12-month prevalence for major depressive disorder in Switzerland, based upon the telephone survey, was 4.4% (95% confidence interval [CI], 4.0-4.8). For the subset of participants who completed the additional postal survey the estimate was comparable (4.2%; 95% CI, 3.7-4.6). Correlations among the social relationship and depression variables were low to moderate suggesting that the social relationship elements are largely independent (see Table 2).

**Table 2:** Correlations among social relationship variables, major depressive disorder, and depressive symptoms, Swiss health survey, 2007

	**1.**	**2.**	**3.**	**4.**	**5.**	**6.**	**7.**
1. Major depressive disorder	-						
2. Depressive symptoms	0.29	-					
3. Confidant	-0.06	-0.10	-				
4. Tangible support	-0.08	-0.14	0.26	-			
5. Unmet support	0.11	0.21	-0.10	-0.13	-		
6. Loneliness	0.22	0.32	-0.07	-0.12	0.27	-	
7. Married	-0.06	-0.08	-0.11	-0.02	-0.08	-0.22	-
8. Social contact frequency	-0.06	-0.10	0.19	0.20	-0.08	-0.08	-0.04

Twelve-month major depressive disorder was assessed with the Composite International Diagnostic Interview short form [38]. Depressive symptoms experienced over the last two weeks were assessed with the Depression Screening Questionnaire [39]. All parametric correlations incorporated the sample weights. The correlation with depressive symptoms is Spearman’s rho. Because of the large sample size all correlations are statistically significant at *p* < 0.05.

### Association of social relationships with 12-month depressive disorder

In bivariate analyses each of the six social relationship variables was associated with major depressive disorder in the expected direction. When all six social relationship variables were entered simultaneously only loneliness, unmet support, and the fourth quintile of social contacts remained significant (Table 3). These patterns persisted when sociodemographic variables were entered, with marital status becoming significant in that model. Loneliness had the strongest association across all models and when we excluded loneliness from the regression the magnitude of association for all other social support variables increased and were statistically significant except for the second and fifth quintiles of the social contacts variable.

**Table 3:** **Incidence rate ratios (95**% **CI) predicting depression with social relationships, Swiss health survey, 2007**

	**Social relationship predictors individually**		**Adjusted for other social relationship variables**		**Further adjusted for sociodemographics**		**Loneliness omitted**	
	**Major depression**	**Depressive symptoms**	**Major depression**	**Depressive symptoms**	**Major depression**	**Depressive symptoms**	**Major depression**	**Depressive symptoms**
Loneliness								
Never	Referent	-	-	-	-	-	-	-
Sometimes	**3.40** (2.58, 4.48)	**1.66** (1.58, 1.73)	**2.94** (2.13, 4.05)	**1.56** (1.49, 1.64)	**2.68** (1.95, 3.68)	**1.55** (1.48, 1.63)	-	-
Quite or very often	**16.4** (12.4, 21.7)	**3.12** (2.87, 3.38)	**11.0** (7.28, 16.5)	**2.58** (2.34, 2.85)	**9.97** (6.84, 14.5)	**2.39** (2.18, 2.63)	-	-
Confidant	**0.44** (0.30, 0.65)	**0.69** (0.64, 0.74)	0.82 (0.53, 1.27)	**0.86** (0.79, 0.93)	0.75 (0.48, 1.18)	**0.90** (0.82, 0.98)	**0.59** (0.38, 0.92)	**0.86** (0.79, 0.94)
Unmet support	**2.74** (2.15, 3.51)	**1.56** (1.49, 1.64)	**1.41** (1.04, 1.90)	**1.27** (1.20, 1.34)	**1.40** (1.04, 1.88)	**1.28** (1.21, 1.35)	**2.11** (1.63, 2.73)	**1.47** (1.39, 1.54)
Tangible support	**0.38** (0.28, 0.53)	**0.64** (0.60, 0.69)	0.77 (0.53, 1.11)	**0.83** (0.77, 0.89)	0.76 (0.52, 1.12)	**0.83** (0.77, 0.90)	**0.58** (0.40, 0.84)	**0.78** (0.72, 0.84)
Social contact								
0-9	-	-	-	-	-	-	-	-
10-12	**0.65** (0.45, 0.94)	**0.82** (0.76, 0.89)	0.87 (0.59, 1.29)	**0.91** (0.84, 0.98)	0.87 (0.58, 1.29)	**0.92** (0.85, 0.99)	0.78 (0.54, 1.13)	**0.90** (0.83, 0.98)
13-14	**0.53** (0.37, 0.77)	**0.77** (0.72, 0.83)	0.75 (0.50, 1.12)	**0.88** (0.81, 0.95)	0.74 (0.48, 1.12)	**0.90** (0.83, 0.97)	**0.66** (0.44, 0.97)	**0.87** (0.80, 0.95)
15-16	**0.38** (0.25, 0.58)	**0.70** (0.64, 0.75)	**0.56** (0.35, 0.90)	**0.81** (0.74, 0.88)	**0.56** (0.34, 0.91)	**0.83** (0.76, 0.91)	**0.50** (0.31, 0.80)	**0.81** (0.74, 0.89)
17-21	**0.49** (0.30, 0.78)	**0.69** (0.63, 0.75)	0.77 (0.47, 1.27)	**0.81** (0.74, 0.88)	0.76 (0.45, 1.28)	**0.81** (0.74, 0.89)	0.65 (0.39, 1.08)	**0.78** (0.71, 0.86)
Married/cohabiting	**0.56** (0.45, 0.71)	**0.89** (0.85, 0.93)	0.80 (0.63, 1.02)	1.01 (0.96, 1.05)	**0.75** (0.59, 0.95)	0.97 (0.92, 1.01)	**0.56** (0.45, 0.71)	**0.87** (0.82, 0.91)
Sociodemographics								
Age								
18-24					Referent	-	-	-
25-34					0.91 (0.54, 1.55)	1.03 (0.93, 1.15)	1.07 (0.63, 1.81)	1.07 (0.97, 1.20)
35-44					1.13 (0.67, 1.91)	1.04 (0.95, 1.15)	1.25 (0.74, 2.11)	1.08 (0.97, 1.19)
45-54					1.07 (0.62, 1.84)	1.08 (0.97, 1.20)	1.17 (0.68, 2.02)	1.10 (0.98, 1.23)
55-64					0.76 (0.44, 1.31)	**1.11** (1.00, 1.24)	0.83 (0.48, 1.43)	**1.12** (1.01, 1.25)
65+					**0.32** (0.18, 0.60)	**1.21** (1.09, 1.33)	**0.31** (0.16. 0.58)	**1.16** (1.05, 1.29)
Male gender					0.84 (0.66, 1.07)	**0.91** (0.87, 0.95)	**0.75** (0.58, 0.97)	**0.88** (0.84, 0.92)
Education								
Mandatory					Referent	-	-	-
Secondary					0.83 (0.59, 1.18)	0.97 (0.89, 1.05)	0.73 (0.51, 1.04)	0.93 (0.86, 1.01)
High school					0.77 (0.45, 1.32)	0.93 (0.83, 1.03)	0.64 (0.37, 1.10)	**0.89** (0.79, 0.99)
University					0.86 (0.57, 1.30)	**0.87** (0.79, 0.96)	0.73 (0.48, 1.12)	**0.83** (0.75, 0.91)
Regional language								
German					Referent	-	-	-
French					0.99 (0.78, 1.27)	**1.32** (1.26, 1.39)	1.09 (0.85, 1.40)	**1.35** (1.28, 1.41)
Italian					0.98 (0.66, 1.45)	**1.22** (1.12, 1.33)	1.10 (0.73, 1.65)	**1.26 **(1.15, 1.37)
Nationality								
Swiss					Referent	-	-	-
Italian					1.28 (0.75, 2.21)	**1.25** (1.05, 1.49)	1.45 (0.80, 2.62)	**1.28** (1.07, 1.52)
Other					1.09 (0.75, 1.58)	1.01 (0.94, 1.09)	1.05 (0.73, 1.52)	1.01 (0.93, 1.09)

Twelve-month major depression was assessed with the Composite International Diagnostic Interview (CIDI) short form [38]. Depressive symptoms experienced over the last two weeks were assessed with the Depression Screening Questionnaire [39]. Incidence rate ratios for major depressive disorder were estimated using Poisson regression with robust standard errors [44]. Incidence rate ratios for depressive symptoms were estimated using negative binomial regression. Confidence intervals (CI) that do not include 1.0 are statistically significant at *p* < = 0.05 and are in **bold**. Confidant and tangible support variables have three levels but have been scaled to 0, 0.5, 1.0 in order to increase comparability with binary predictors. CI = confidence interval.

### Association of social relationships with depressive symptoms

All social relationship variables were associated with depressive symptoms when analyzed individually, which is in line with the depressive episode analyses. In contrast to depressive episodes analyses, marital status was not significantly associated with depressive symptoms when all six variables were entered simultaneously. Although loneliness and unmet support had the strongest associations, each social contact frequency category was inversely associated with depressive symptoms, with the benefit of increased social contact frequency leveling off at the two highest quintiles. This pattern of independence among social relationship variables persisted following statistical adjustment for sociodemographic variables (Table 3). When we excluded loneliness from the regression all other social relationship variables, including marital status, were significant and were of somewhat larger magnitude (Table 3).

We conducted exploratory analyses to partition the very large association of loneliness with depressive symptoms. We estimated mean depressive symptoms within the three loneliness categories to allow a more clinically meaningful interpretation. Mean depressive symptoms differed strongly according to loneliness, increasing significantly from *never* (*M* = 2.6 [95% CI, 2.6 – 2.7]) to *sometimes* (*M* = 4.4 [95% CI, 4.2 – 4.5]) to *quite/very often* (*M* = 8.2 [95% CI, 7.6 – 8.9]).

## Discussion

We found that each social relationship dimension representing social support and social integration was associated with both major depressive episodes and depressive symptoms. These associations were more likely to persist in multivariate models with continuous depressive symptoms as an outcome variable as compared to the outcome of a binary major depressive episode. Although the social relationship variables are conceptually related they appear to tap unique aspects of the social environment as evidenced by their small bivariate intercorrelations and their independent associations when entered jointly into regression models predicting depression. Social relationship variables were more consistently associated with continuous depressive symptoms, which is not surprising given statistical difficulties predicting rare outcomes such as a recent major depressive episode.

Loneliness had the strongest association with depressive episodes, with those reporting being lonely *sometimes* having greater rates of a depressive episode and those reporting feeling lonely *quite* or *very often* having dramatically higher rates relative to those who reported *never* feeling lonely. Our data also showed that being unmarried and having unmet support needs were independently associated with a major depressive episode when controlling for loneliness. These patterns suggest that emotional support facets have distinct associations with recent major depressive episode incidence as assessed by a structured interview.

As with depressive episodes, the subjective perception of loneliness was the strongest correlate of depressive symptoms. Moreover, all social relationship variables except for marital status were associated with depressive symptoms. The large loneliness regression coefficients observed in these data from Switzerland correspond well with those of older adults in the US [31] (depressive symptoms) and the general population in Australia [23] (major depression)^a^. This emerging evidence, combined with recent evidence that loneliness has adverse consequences for physical as well as mental health [13,49], justifies increased scholarly attention to loneliness.

In addition to loneliness, being less socially integrated and not having a confidant were independently associated with depressive symptoms. This suggests that it is useful to assess indicators of both *social* and *emotional* loneliness, the loneliness elements theorized to correspond with low social integration and lacking a confidant [50,51]. Also notable is that having a confidant is important, but having a confidant is not equivalent to meeting one’s need to confide about problems. Broader social resources, such as having others available to help with daily activities, are associated with depression on a magnitude similar to having a confidant (see [24] for a similar finding). This is consistent with theoretical predictions because the availability of such support, by signaling concern for ones’ welfare, could satisfy the human need for belonging [14].

The inverse associations we observed between depression and social relationship quality and quantity replicates other studies [23-26,31,34,52] and provides confirmatory (but not definitive) evidence that social relationships are fundamental to human well-being [12,14,15,53]. Contrary to the assertion that only subjective perceptions of relationship quality are important for human well-being [23], we found independent associations of quantitative social relationship indicators for major depressive episodes (marital status) and for depressive symptoms (social contact frequency). The literature thus far is inconsistent - marital status was unrelated to incident major depressive disorder in another population-based study [34] whereas a different social relationship dimension, the absence of contact with close friends, was associated with major depressive disorder [35]. Depressive symptoms and social contacts are also inconsistently related [26,54]. Recent work shows that group identification, rather than social participation per se, captures the salutary influence of social participation on depressive symptoms [55]. Thus, including group identification assessments in future research may clarify these patterns. More broadly, examining disaggregated social relationship domains will advance theory and facilitate comparison across studies that often utilize conceptually similar but empirically distinct operational definitions.

The strong associations observed for this brief set of social relationship indicators in the Swiss National Health Survey suggests that public health surveys can assess theoretically and empirically important social relationship resources without excessive cost or participant burden. For example, the size and relative magnitude of regression coefficients for our single loneliness item were comparable to those estimated using long (20 items) and short (3 items) loneliness assessments in another study [31]. Brief measures of social integration and social support are strongly associated with mental [32,53] and physical [56-58] health and could be added where resources and interests permit. Adopting more diverse social relationship assessments in community epidemiology can inform and complement parallel efforts in clinical epidemiology to advance our understanding of the social resources necessary for optimal mental health.

### Study strengths and limitations

The present study utilized a large, probability sample that represented young adults, a group particularly vulnerable to major depression. We assessed depression with interview and questionnaire methods and we evaluated their association with a large number of variables representing two broad, theoretically important social relationship domains. While not a focus of the present study, we also incorporated “upstream” social and contextual measures in our analyses to more precisely characterize the unique contribution of social relationships [15]. To our knowledge this is one of a handful of nationally representative studies in this area and the first one from Europe including adults 18 years and older. Although Switzerland has a relatively low base rate of loneliness relative to other countries [59] the associations we observed are consistent with prior research utilizing more restricted samples.

This study is also subject to a number of limitations. There are several ways to define and measure social relationships and our study did not assess potentially important domains such as social strain [34], relationship reciprocity or marital satisfaction. The cross-sectional design of our study cannot address the causal direction of the association, i.e., whether lower social support and less social integration cause depression or the other way around. There is evidence for both perspectives. For example, we know that humans are motivated to form and maintain social attachments [14], that social exclusion is painful [60], and that theory predicts that depressive symptoms should be a consequence of low social relationship resources [12,15]. Longitudinal studies confirm that greater social relationship resources are prospectively associated with fewer depressive symptoms [19,33,54,61], less probable incident depression [32] and better recovery from major depression even when controlling for initial depression levels [20,62]. Conversely, among adolescents there is evidence that depression is associated with subsequent increases in loneliness [63] and dysfunctional attributions regarding social resources can elicit depression [64] but may resolve after the depressive episode [65]. Low self esteem or low social skills are etiologically relevant for both loneliness and depression [66-68]. Of course, one can also be depressed without feeling lonely [16] and vice-versa. While acknowledging these reciprocal possibilities it is reasonable to conceptualize low social relationship resources as determinants of depression [69] and as plausible targets for intervention [70]. Future studies with longitudinal measurements of depression and social relationships will help clarify the presence and magnitude of these reciprocal influences (cf. [19,71]).

## Conclusion

We found that both subjective and quasi-objective social relationship measures were independently associated with clinical depression and depressive symptoms in a nationally representative sample of Swiss adults. Perceived loneliness and unmet support were most strongly related to each outcome in multivariate models, and loneliness attenuated the association of qualitative and quantitative social relationship variables. The magnitude and significance of social relationship associations were sensitive to the type of depression outcome, in that social relationships were more consistently associated with depressive symptoms versus recent major depressive episodes. This study also provides evidence for these associations in young adulthood, expanding the literature establishing the importance of the social environment to psychological well-being.

## Endnote

^a^The unexponentiated negative binomial regression coefficient for a single loneliness item in our fully adjusted model was 0.41. The most similar coefficient reported in Reference 31, Table 2 was 0.40. The odds ratio for loneliness/social isolation predicting major depression in Reference 23 was 21.42. This value was close to the point estimate and within the 95% confidence interval for the odds of depression for the highest loneliness category in the present data.

## Abbreviations

CIDI-SF: Composite international diagnostic interview-short form; OLS: Ordinary least squares; IRR: Incidence rate ratio; CI: Confidence interval.

## Competing interests

The authors declare that they have no competing interests.

## Authors’ contributions

SB analyzed the data and drafted the manuscript. SB, JB and NM conceptualized the research question, JB and NM edited the manuscript. All authors read and approved the final version of the manuscript.

## Pre-publication history

The pre-publication history for this paper can be accessed here:

http://www.biomedcentral.com/1471-2458/14/273/prepub
